# Empyema necessitans complicating a mild COVID‐19 infection in a young immunocompetent patient

**DOI:** 10.1002/ccr3.7598

**Published:** 2023-06-15

**Authors:** Sierra Sullivan, Jesus Davalos, Belaal Sheikh, Mahmoud Abdelnabi

**Affiliations:** ^1^ Internal Medicine Department Texas Tech University Health Science Center Lubbock Texas USA; ^2^ Pulmonary and Critical Care Division, Internal Medicine Department Texas Tech University Health Science Center Lubbock Texas USA

**Keywords:** COVID‐19, empirical antibiotic therapy, empyema necessitans, immunocompetent patient, surgical drainage

## Abstract

COVID‐19 infection can be complicated by secondary bacterial infections including pneumonia and empyema resulting in worse outcomes. Empyema management includes empirical antibiotic therapy and drainage with a favorable prognosis in most cases.

**Abstract:**

Empyema necessitans is a rare complication of poorly or uncontrolled empyema thoracis resulting in the dissection of pus through the soft tissues and skin of the chest wall resulting in a fistula between the pleural cavity and the skin. Previous reports indicate that secondary bacterial pneumonia can complicate the course of a COVID‐19 infection even in immunocompetent patients resulting in worse outcomes. Management of empyema includes empirical antibiotic therapy and drainage with a favorable prognosis in most cases.

## Graphical abstract

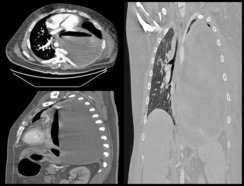

## 1 CASE PRESENTATION

**FIGURE 1:**
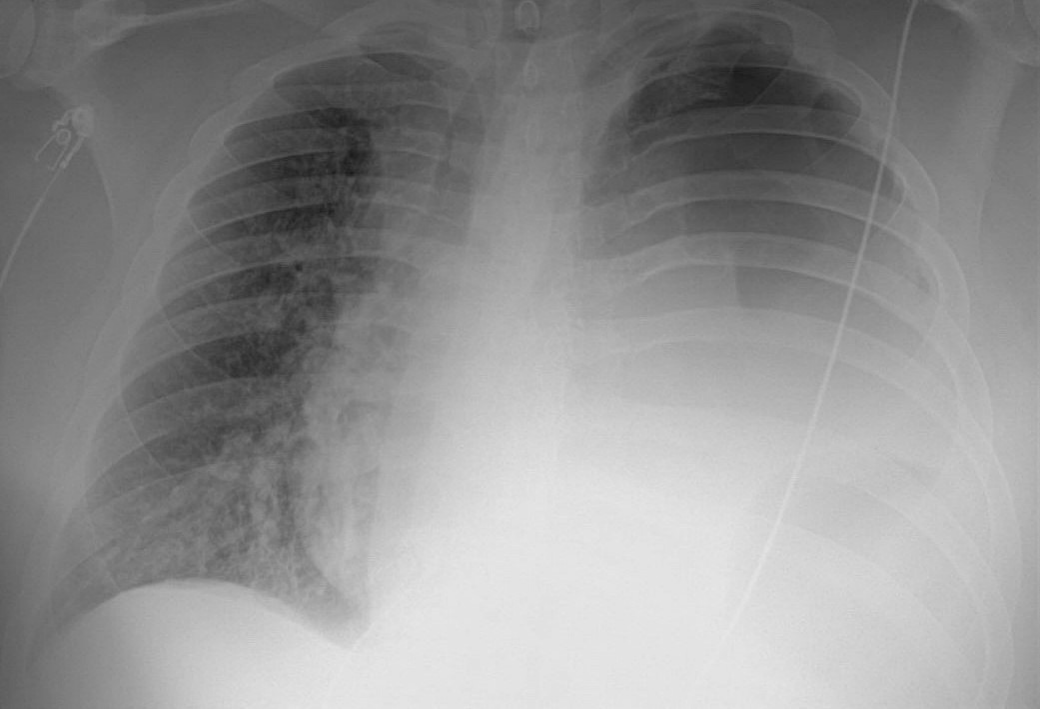
Chest X‐ray showed opacified left hemithorax possibly by pleural effusion with a mediastinal shift.

**FIGURE 2:**
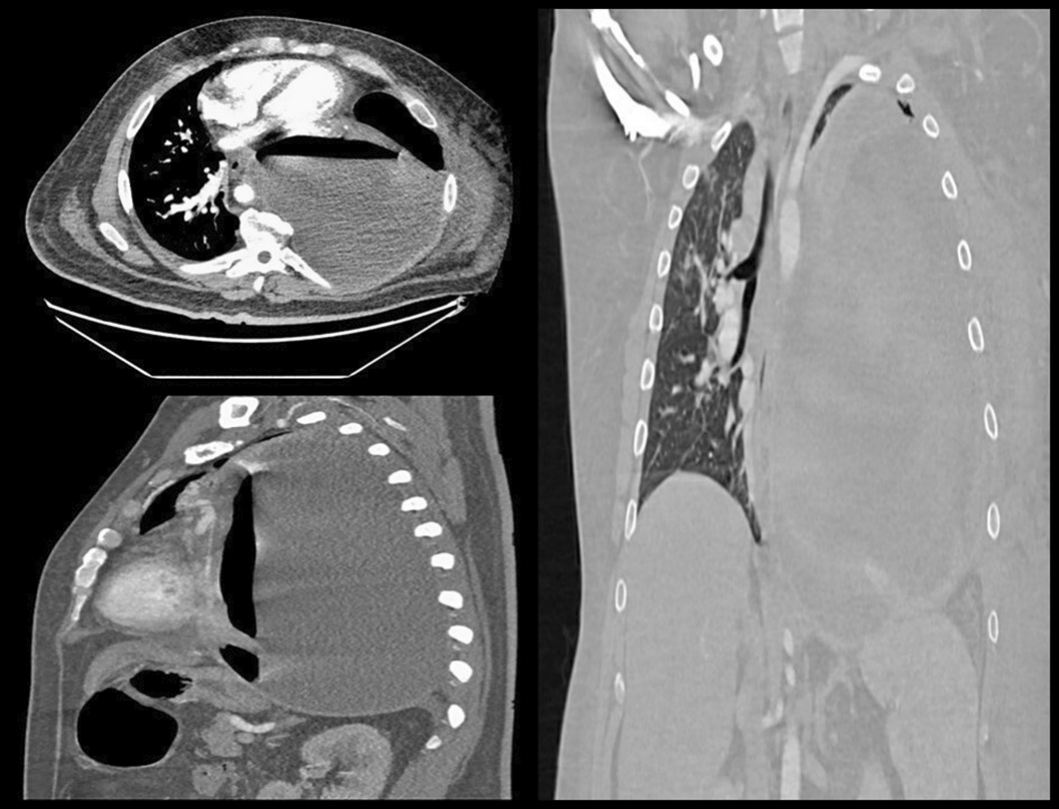
Large cavitary lesion in the left lung containing a large volume of fluid with air‐fluid level exerting a mass effect on mediastinal structures, large left hydropneumothorax, and multifocal airspace opacities in the right lung.

**FIGURE 3:**
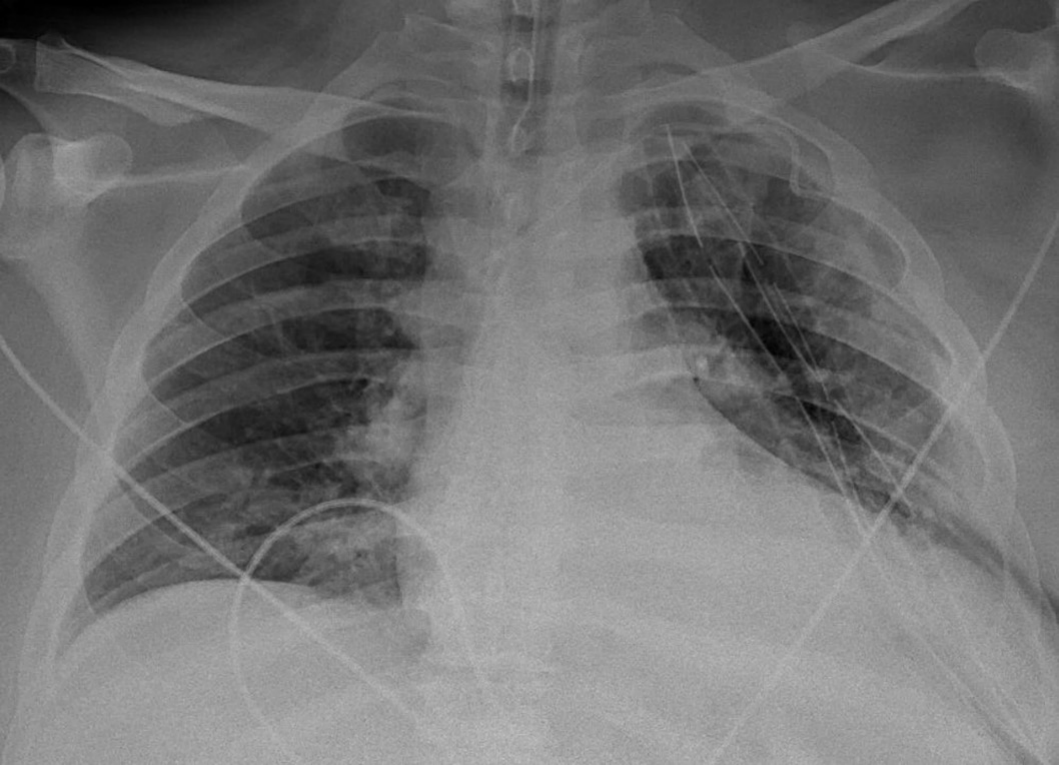
Chest X‐ray showed an overall improvement of the left lung with left‐sided chest tubes being in place.

A man in his 30 s with a history of obesity class II presented as a referral from another hospital complaining of a 1‐month history of cough, shortness of breath, and a 2‐week history of tender left‐sided chest mass after having flu‐like symptoms for a few days following his exposure to a family member who tested positive for COVID‐19. He initially developed a dry cough that progressed to a productive cough with yellow sputum associated with worsening shortness of breath, left‐sided chest pain, fatigue, malaise, and pain and left‐sided warm tender mass. On general examination, he was distressed, leaning forwards with pursed lip breathing with significant accessory muscle use. A left‐sided chest wall mass that was warm and tender to palpation with fluctuance. He was afebrile with a temperature of 98.2 °F, tachycardic with a heart rate of 120 beats per minute and blood pressure of 142/88 mmHg, tachypneic with a respiratory rate of 27 per minute and oxygen saturation of 98% on 6 liters (L) nasal cannula. Lung auscultation showed diminished breath sounds on the left lung. Initial laboratory workup was remarkable for leukocytosis with neutrophilia (WBCs of 15.6 × 109/L with 90% neutrophils) and lactic acidosis (Lactic acid level of 1.8 mmol/L). A respiratory viral panel by polymerase chain reaction (PCR) was negative, however, COVID‐19 2 IgG antibody was positive indicating a previous infection. Chest X‐ray showed opacified left hemithorax possibly by pleural effusion with mediastinal shift (Figure 1). Chest computed tomography (CT) showed a large cavitary lesion in the left lung containing a large volume of fluid with air‐fluid level exerting a mass effect on mediastinal structures, large left hydropneumothorax, and multifocal airspace opacities in the right lung (Figure 2). He was started empirically on intravenous (IV) vancomycin and cefepime. A left posterolateral thoracotomy with chest wall debridement and empyema decortication with left chest tube placement. Follow‐up chest X‐ray showed an overall improvement of the left lung with left‐sided chest tubes being in place (Figure 3). Empyema fluid cultures showed pan‐sensitive *Streptococcus anginosus* and *Streptococcus constellatus* and blood cultures were negative. His condition improved gradually, his chest tubes were removed, and he was discharged to continue a course of 6 weeks of amoxicillin/clavulanic acid in outpatient settings. Empyema necessitans is a rare complication of poorly or uncontrolled empyema thoracis characterized by the dissection of pus through the soft tissues and skin of the chest wall. The pus collection bursts and communicates with the exterior, forming a fistula between the pleural cavity and the skin.^1^ The most commonly associated pathogens are *Mycobacterium tuberculosis* and *Actinomyces israelii*.^2^ The most common non‐tubercular etiologies are *Staphylococcus* species.^1^ Other etiologies include *Pneumococci*, *Escherichia coli*, *Pseudomonas*, *Klebsiella*, and anaerobes.^1^ Previous reports indicate that secondary bacterial pneumonia or less commonly empyema can complicate the course of a COVID‐19 infection even in immunocompetent patients resulting in worse outcomes.^3^, ^4^, ^5^ Management includes empirical antibiotic therapy and drainage; decortication might be considered in patients with organized or established fibrothorax or evidence of trapped lung alternatively intrapleural tissue plasminogen activator with DNase can be utilized in non‐operable cases. Long‐term prognosis is overall favorable in cases of empyema with a higher risk of mortality in patients who required open surgery or decortication.^6^

## AUTHOR CONTRIBUTIONS

**Sierra Sullivan:** Writing – original draft; writing – review and editing. **Jesus Davalos:** Writing – original draft; writing – review and editing. **Belaal Sheikh:** Writing – original draft; writing – review and editing. **Mahmoud Abdelnabi:** Writing – original draft; writing – review and editing.

## CONFLICT OF INTEREST STATEMENT

None declared.

## CONSENT

Patient consent has been signed and collected in accordance with the journal's patient consent policy.

## INFORMED CONSENT

The authors have obtained written informed consent from the patient to publish his case.

## Data Availability

All case‐related data are available as part of the article and no additional source data are required.

## References

[1] Yauba M , Ahmed H , Imoudu I , Yusuf M , Makarfi H . Empyema necessitans complicating pleural effusion associated with proteus species infection: a diagnostic dilemma. Case Rep Pediatr. 2015;2015:1‐4. 10.1155/2015/108174 PMC4393920 25893125

[2] Akgül AG , Örki A , Örki T , Yüksel M , Arman B . Approach to empyema necessitatis. World J Surg. 2011;35:981‐984. 21404081 10.1007/s00268-011-1035-5

[3] Shafran N , Shafran I , Ben‐Zvi H , et al. Secondary bacterial infection in COVID‐19 patients is a stronger predictor for death compared to influenza patients. Sci Rep. 2021;11:1‐8. 34135459 10.1038/s41598-021-92220-0 PMC8209102

[4] Ayad S , Gergis K , Elkattawy S , et al. Loculated empyema and SARS‐CoV‐2 infection: a report of two cases and review of the literature. Eur J Case Rep Intern Med. 2021;8(7):1‐7. 10.12890/2021_002706 PMC8336741 34377699

[5] Yarlagadda K , Mi K , Sendil S , Koons CL , Komanduri S , Cinicola JT . A 31‐year‐old man with COVID‐19‐associated empyema and lupus anticoagulant. Amer J Cas Rep. 2020;21:e926623‐1. 10.12659/AJCR.926623 PMC7458696 32807764

[6] Semenkovich TR , Olsen MA , Puri V , Meyers BF , Kozower BD . Current state of empyema management. Ann Thorac Surg. 2018;105:1589‐1596. 29550205 10.1016/j.athoracsur.2018.02.027 PMC5964038

